# Retraction Note: Cost effective liquid phase exfoliation of MoS_2_ nanosheets and photocatalytic activity for wastewater treatment enforced by visible light

**DOI:** 10.1038/s41598-024-67387-x

**Published:** 2024-07-22

**Authors:** Dhirendra Sahoo, Birendra Kumar, Jaivardhan Sinha, Subhasis Ghosh, Susanta Sinha Roy, Bhaskar Kaviraj

**Affiliations:** 1 Department of Physics, School of Natural Sciences, Shiv Nadar University, NH91, Gautam Budh Nagar, Greater Noida, Uttar Pradesh 201314 India; 2 School of Physical Sciences, Jawaharlal Nehru University, New Delhi, 110067 India; 3 Department of Physics and Nanotechnology, SRM Institute of Science and Technology, Kattankulathur, Kancheepuram, 603203 India

Retraction of: *Scientific Reports* 10.1038/s41598-020-67683-2, published online 01 July 2020

The Editors have retracted this Article.

After publication of this Article, concerns were raised about the XRD data reported in Figure 3. Specifically, there appear to be areas of repeating noise between the 0.3 and 0.4 mg/ml traces, and there are irregular patterns in each of the the 0.12 and 0.08 mg/ml traces. The Authors provided raw data for this figure upon request by the Editors, but further discrepancies were observed between this raw data and the data reported in the published Figure 3. The Editors no longer have confidence in the reliability of the findings and conclusions of this article.

Dhirendra Sahoo, Bhaskar Kaviraj, and Subhasis Ghosh did not explicitly state whether they agree or disagree with this retraction. The remaining authors did not respond to correspondence from the Editors about this retraction.

